# Supplementary material for: Impacts of community forestry on forest condition: Evidence from Sri Lanka’s intermediate zone
Source: PLoS One. 2020 Sep 30;15(9):e0239405. doi: 10.1371/journal.pone.0239405 (PMC7526917; doi:10.1371/journal.pone.0239405)
Supplement: S1 Table — (DOCX) [file pone.0239405.s001.docx]

**S1 Table.** List of woody species recorded in semi mixed evergreen forest in the Intermediate Zone

| **Scientific name** | **Family** | **CMBs** | **SMBs** |
| --- | --- | --- | --- |
| 1. *Acacia caesia* | FABACEAE | X | X |
| 1. *Acacia leucophloea* | FABACEAE | X | X |
| 1. *Acronychia peduncidata,* | RUTACEAE |  | X |
| 1. *Actinodaphne stenophylla* | LAURACEAE |  | X |
| 1. *Albizia odoratissima* | FABACEAE | X | X |
| 1. *Alphonsea zeylanica* | ANNONACEAE |  | X |
| 1. *Alseodaphne semecarpifolia* | LAURACEAE | X | X |
| 1. *Alstonia scholaris* | APOCYNACEAE |  | X |
| 1. *Alstonia macrophylla* | APOCYNACEAE | X | X |
| 1. *Anamirta cocculus* | MENISPERMACEAE | X | X |
| 1. *Anodendron manubriatum* | APOCYNACEAE | X | X |
| 1. *Anogeissus latifolius* | COMBRETACEAE |  | X |
| 1. *Antidesma thwaitesianum* | PHYLLANTHACEAE |  | X |
| 1. *Artabotrys hexapetalus* | ANNONACEAE |  | X |
| 1. *Artocarpus nobilis* | MORACEAE |  | X |
| 1. *Azadirachta indica* | MELIACEAE | X | X |
| 1. *Bauhinia tomentosa* | FABACEAE | X | x |
| 1. *Benkara malabarica* | RUBIACEA |  | X |
| 1. *Bndelia retusa* | EUPHORBIACEAE | X | X |
| 1. *Breynia retusa* | PHYLLANTHACEAE | X | X |
| 1. *Butea monosperma* | FABACEAE |  | X |
| 1. *Canarium zeylanicum* | BURSERACEAE |  | X |
| 1. *Carey a arborea* | LECYTHIDACEAE | X | X |
| 1. *Cassia auriculata* | FABACEAE | X | X |
| 1. *Catunaregam spinosa* | RUBIACEA | X | X |
| 1. *Coryota urens* | PALMAE |  | X |
| 1. *Calophyllum calaba* | CLUSIACEAE |  | X |
| 1. *Chloroxylon swietenia* | RUTACEAE | X | X |
| 1. *Chukrasia tabularis* | MELIACEAE | X | X |
| 1. *Cinnamomum dubium* | LAURACEAE |  | X |
| 1. *Cinnamomum zeylanicum* | LAURACEAE |  | X |
| 1. *Cipadessa baccifera,* | MELIACEAE | X | X |
| 1. *Clausena indica* | RUTACEAE | X | X |
| 1. *Cleistocalyx operculatus* | MYRTACEAE |  | X |
| 1. *Clerodendrum inerme* | LAMIACEAE | X | X |
| 1. *Crateva adansonii* | LYTHRACEAE | X | X |
| 1. *Croton aromaticus* | EUPHORBIACEAE | X | X |
| 1. *Derris scandens* | FABACEAE |  | X |
| 1. *Dimocarpus longan,* | SAPINDACEAE | X | X |
| 1. *Diplodiscus verrucosus* | TILIACEAE |  | X |
| 1. *Diospyros chaetocarpa* | EBENACEA |  | X |
| 1. *Diospyros insignis* | EBENACEA |  | X |
| 1. *Diospyros ovalifolia,* | EBENACEA |  | X |
| 1. *Diyaminauclea zeylanica* | RUBIACEA |  | X |
| 1. *Drypetes sepiaria* | EUPHORBIACEAE | X | X |
| 1. *Ehretia microphylla* | BORAGINACEAE | X | X |
| 1. *Entada pusaetha* | FABACEAE |  | X |
| 1. *Erythrina vanegata* | FABACEAE | X | X |
| 1. *Eugenia bracteata* | MYRTACEAE |  | X |
| 1. *Euphorbia antiquorum* | EUPHORBIACEA |  | X |
| 1. *Ficus amplissima* Smith | MORACEAE |  | X |
| 1. *Ficus arnottiana* | MORACEAE | X | X |
| 1. *Ficus benghalensis* | MORACEAE | X | X |
| 1. *Ficus tsjahela* | MORACEAE |  | X |
| 1. *Ficus racemosa* | MORACEAE |  | X |
| 1. *Ficus exasperata* | MORACEAE | X | X |
| 1. *Ficus hispida* | MORACEAE |  | X |
| 1. *Filicum decipiens* | SAPINDACEAE |  | X |
| 1. *Flueggea leucopyrus* | EUPHORBIACEA | X | X |
| 1. *Garcinia morella* | CLUSIACEAE |  | X |
| 1. *Gliricidia septum* | FABACEAE | X | X |
| 1. *Glochidion stellatum* | PHYLLANTHACEAE |  | X |
| 1. *Glochidion zeylanicum* | PHYLLANTHACEAE | X | X |
| 1. *glycosmis pentaphylla* | RUTACEAE | X | X |
| 1. *Gmelina arborea* | VERBENACEAE | X | X |
| 1. *Goniothalamus thiuaitesii* | ANNONACEAE |  | X |
| 1. *Grewia damine* | TlLIACEAE | X | X |
| 1. *Grewia helicterifolia* | TILIACEAE |  | X |
| 1. *Grewia polygama* | MALVACEAE |  | X |
| 1. *Haldina cordifolia* | RUBIACEA | X | X |
| 1. *Helicteres isor* | STERCULIACEA | X | X |
| 1. *Hiptage benghalensis* | MALPIGHIACEAE | X | X |
| 1. *Litsea iteodaphne* | LAURACEA |  | X |
| 1. *Litsea glutinosa* | LAURACEA | X | X |
| 1. *Madhuca longifolia,* | SAPOTACEAE |  | X |
| 1. *Macaranga indica* | EUPHORBIACEAE | X | X |
| 1. *Macaranga peltata,* | EUPHORBIACEAE | X | X |
| 1. *Mallotus philippensis* | EUPHORBIACEAE |  | X |
| 1. *Mallotus tetracoccus* | EUPHORBIACEAE |  | X |
| 1. *Manilkara hexandra* | SAPOTACEAE | X | X |
| 1. *Meliosma pinnata* | SABIACEAE |  | X |
| 1. *Memecylon angustifolium,* | MELASTOMATACEAE |  | X |
| 1. *Mesua nagassarium* | CLUSIACEAE | X | x |
| 1. *Michelia champaca* | MAGNOLIACEAE | X | X |
| 1. *Micromelium ceylanicum* | RUTACEAE | X | X |
| 1. *Microcos paniculata* | MALVACEAE | X | X |
| 1. *Miliusa tomentosa* | ANNONACEAE |  | X |
| 1. *Myristica dactyloides* | MYRISTICACEAE | X | X |
| 1. *Neolitsea cassia* | LAURACEAE | X | X |
| 1. *Nothopegia beddomei* | ANACARDIACEAE | X | X |
| 1. *Pagianiha dichotom* | APOCYNACEAE |  | X |
| 1. *Paramignya monophylla* | RUTACEAE |  | X |
| 1. *Phoenix farinifera* | PALMAE | X | X |
| 1. *Phyllanthus emblica* | EUPHORBIACEAE | X | X |
| 1. *Phyllanthus polyphyllus* | EUPHORBIACEAE | X | X |
| 1. *Polyalthia korinti* | ANNONACEAE | X | x |
| 1. *Pometia eximia* | SAPINDACEAE | X | X |
| 1. *Pongamia pinnata* | FABACEAE | X | X |
| 1. Premna wightiana | VERBENACEA |  | X |
| 1. *Premna tomentosa* | VERBENACEAE | X | X |
| 1. *Psidium guineense* | MYRTACEAE | X | X |
| 1. *Pterocarpus marsupium* | FABACEAE | X | X |
| 1. *Pterospertmim suberifolium* | STERCULIACEAE | X | X |
| 1. *Rejoua dichotoma* | APOCYNACEAE | X | X |
| 1. *Sapindus emarginatiis* | SAPINDACEAE |  | X |
| 1. *Schleichera oleosa,* | SAPINDACEAE |  | X |
| 1. *Semecarpus gardneri* | ANACARDIACEAE |  | X |
| 1. *Syzygium neesianum* | MYRTACEAE |  | X |
| 1. *Syzygium gardneri* | MYRTACEAE | X | X |
| 1. *Sterculia foetida* | MALVACEAE | X | x |
| 1. *Streblus taxoides* | MORACEAE | X | X |
| 1. *Streblus asper* | MORACEAE |  | X |
| 1. *Schefflera stellata* | ARALIACEAE |  | X |
| 1. *Tarenna asiatica* | RUBIACEA | X | X |
| 1. *Terminalia arjuna* | COMBRETACEAE |  | X |
| 1. *Terminalia bellirica* | COMBRETACEAE | X | X |
| 1. *Terminalia chebula* | COMBRETACEAE |  | X |
| 1. *Thespesia populnea* | MALVACEAE | X | X |
| 1. *Trema orientalis,* | ULMACEAE |  | X |
| 1. *Turpinia malabarica* | STAPHYLEACEAE | X | X |
| 1. *Vernonia arborea* | COMPOSITAE |  | X |
| 1. *Vitex altissima* | LAMIACEAE | X | X |
| 1. *Vitex negundo* | LAMIACEAE | X | X |
| 1. *Vitex pinnata* | LAMIACEAE | X | X |
| 1. *Wendlandia Toicuspidata* | RUBIACEA |  | X |
| 1. *Woodfordia fruticosa* | LYTHRACEAE |  | X |
| 1. *Ziziphus oenoplia* | RHAMNACEAE | X | X |
