# Supplementary material for: Impacts of community forestry on forest condition: Evidence from Sri Lanka’s intermediate zone
Source: PLoS One. 2020 Sep 30;15(9):e0239405. doi: 10.1371/journal.pone.0239405 (PMC7526917; doi:10.1371/journal.pone.0239405)
Supplement: Attachment — Submitted filename: Rebuttal letter.docx [file pone.0239405.s002.docx]

**Rebuttal letter**

**Reviewer 1**

**Question 1:** Is the manuscript technically sound, and do the data support the conclusions?

**Comment of Reviewer #1:** Partly

**Response:** We are grateful to the first reviewer’s comment. We have modified the original version with technically sound scientific research data that supports the conclusions. Also, our experiment conducted rigorously, with appropriate controls, replication, and sample sizes.

**Question 2.** Has the statistical analysis been performed appropriately and rigorously?

**Comment of Reviewer #1:** Yes

**Response:** We are grateful to the first reviewer’s comment.

**Question 3.** Have the authors made all data underlying the findings in their manuscript fully available?
**Comment of Reviewer #1:** NO

**Response:** Thank you for the helpful comment. In the current version of the manuscript, the data underlying the findings described in the manuscript are fully available without restriction.

**Question 4.** Is the manuscript presented in an intelligible fashion and written in Standard English?
**Comment of Reviewer #1:** NO

**Response:** We are grateful for the comment. In the current version of the manuscript, the language corrected with the help of a professional English editor.

**Question 5.**

**Comment of Reviewer #1:** The manuscript presents a case study on the impacts of the community forestry (CF) program in Sri Lanka on forest conditions (e.g. species diversity, composition and disturbances), applying a before-after control impact (BACI) approach. The study is well-designed and provides interesting and valuable results on how community forest management can affect and even improve forest environments to the benefit of rural communities by enhancing their provision of environmental services and forest products for subsistence needs and markets.

While this manuscript has potential for publication.

(a) Among my major concerns is that it is a case study based on a very small sample area of forest (70 ha)
(b) Clarity of data availability and data used
(c) Specific management activities in community and state forests before and after implementing the CF program,
(d) clarifying the role or proportion of valuable tree species (timber, fruit, etc.) among new species, saplings and seedlings in community and state forests
(e) 4) Discussing the issue of potential spillover effects into state forests from management and policy interventions in community forests.

**Response:** Thank you for the helpful suggestions. We modified the current version of the manuscript as follows

(a)	The current version of the manuscript is revised by increasing the sample size. The new manuscript is based on the data of 9 forest sitess in intermediate zone.

(b)	The current version of the manuscript is revised by making fully available data without restriction.

(c)	The current version of the manuscript is revised by adding the specific management activities in community and state forests before and after implementing the CF program (Conceptual framework; Lines 171-179).

(d)	The current version of the manuscript is revised by adding roll or proportion of valuable tree species (timber, fruit, etc.) in community and state forests (Lines 535-543).

(e)	The current version of the manuscript is revised by adding potential spillover effects into state forests from management and policy interventions in community forests. (Lines 621-630)

**Reviewer 2**

**Question 1:** Is the manuscript technically sound, and do the data support the conclusions?

**Comment of Reviewer #1:** Yes

**Response:** We are grateful to the second reviewer’s comment.

**Question 2.** Has the statistical analysis been performed appropriately and rigorously?

**Comment of Reviewer #2:** Yes

**Response:** We are grateful to your comment.

**Question 3.** Have the authors made all data underlying the findings in their manuscript fully available?
**Comment of Reviewer #2:** Yes

**Response:** Thank you very much for your comment. In the current version of the manuscript, the data underlying the findings described in the manuscript are fully available without restriction.

**Question 4.** Is the manuscript presented in an intelligible fashion and written in Standard English?
**Comment of Reviewer #1:** yes

**Response:** Thank you for the helpful comment.

**Question 5.**

**Comment of Reviewer #2:** I found merit in the detail and comprehensive study, thought on the local level. The manuscript describes impact of community forestry on forest conditions in Kandy District of Sri Lanka using the before-after control impact (BACI). The paper has local impact, even though conclusions can be accepted in general.
The introduction describes the current situation with sufficient literature review. The methodology part describes conceptual framework and data collection on the right way. However subchapter “Data analysis” is very general, short and incomplete. I suggest to add more detail information how authors applied this method on their condition. (e.g. add what statistical significance you calculated later).
Moreover, I suggest to add additional statistical analyses (like data reliability or additional statistical test to improve relevance of data for conclusions).
I highly appreciate that authors collected relevant data to assess the impact of community forestry, which has been discussed and compare with many other researches.
Finally, I recommend manuscript for publishing after minor edits.

**Response:** Thank you for the helpful suggestions. In the current version of the manuscript, the “Data analysis” part was modified by adding more details information on how this method applied in forest condition (Lines 369-375)
