# Supplementary material for: Impacts of community forestry on forest condition: Evidence from Sri Lanka’s intermediate zone
Source: PLoS One. 2020 Sep 30;15(9):e0239405. doi: 10.1371/journal.pone.0239405 (PMC7526917; doi:10.1371/journal.pone.0239405)
Supplement: Attachment — Submitted filename: Response to Reviewers.docx [file pone.0239405.s004.docx]

**Rebuttal letter for manuscript: PONE-D-20-13177**

**Reviewer 1**

**Question 1:** Is the manuscript technically sound, and do the data support the conclusions?

**Comment of Reviewer #1:** Partly

**Response:** We are grateful to the first reviewer’s comment. We have modified the original version with technically sound scientific research data that supports the conclusions. Moreover, our experiment was conducted rigorously, with appropriate controls, replication, and sample sizes.

**Question 2.** Has the statistical analysis been performed appropriately and rigorously?

**Comment of Reviewer #1:** Yes

**Response:** We are grateful to the first reviewer’s comment.

**Question 3.** Have the authors made all data underlying the findings in their manuscript fully available?
**Comment of Reviewer #1:** NO

**Response:** Thank you for the helpful comment. In the current version of the manuscript, the data underlying the findings described in the manuscript are fully available without restriction.

**Question 4.** Is the manuscript presented in an intelligible fashion and written in Standard English?
**Comment of Reviewer #1:** Yes

**Response:** We are grateful to the first reviewer’s comment.

**Question 5.**

**Comment of Reviewer #1:**

This review of the manuscript “Impacts of community forestry on forest condition: Evidence from Sri Lanka's intermediate zone” (PONE-D-20-13177) constitutes the second revision of the previous version “Community forestry in Sri Lanka: Case study on impacts on forest condition” (PONE-D-19-21562). The authors have revised the study and analysis, attending the main issue concerning its applicability for publication in PLOS ONE journal, mainly the sample size of CF sites for its assessment. In the revised version, the sample increases form being a case study of one CF site of 70 ha, to a total of 9 CF sites with greater spatial coverage of mixed semi-evergreen forest in Sri Lanka. Other issues regarding its redaction and greater detail in the results presented, such as the use and value of seedlings, saplings and trees recorded in sampled plots have also been attended. Thus, there is a major improvement to the manuscript and is now acceptable for publication after some modifications and clarifications in the text that require attention.

**Response:** We are grateful to the first reviewer’s beneficial comments. We have modified the original version and have re-submitted a new version by following all of your comments.

(a) Present a more formal and current definition of Community Forest Management (e.g. Charnley and Poe, 2007).

**Response:** We are grateful to this comment. The current version of the manuscript is revised by including the more formal and current definition of CF (line 46-49)

(b)What are the criteria in terms of forest resource access, use and rights, land tenure, govt. institutions, etc. and how are these conditions represented in Sri Lanka?

**Response:** We are grateful to this comment. The manner in which power and rights (i.e., resource access) change from state-managed to community-managed is shown in the conceptual framework (Fig. 1) and line 176-186.

**(c)** In the Methodology section, while the authors have offered a list of specific management interventions undertaken by the FD in the forest before CF, they still do not clearly characterize how management interventions differ (or not) before and after CF implementation within CF and SM forests. The impression is that management interventions occur in CF sites (before and after) and not in SM sites (no management or just protection). Please clarify this in the text, or it could be presented in a simple table.

**Response:** We are grateful to this comment. The current version of the manuscript is revised by adding the main differences of management in community and state forests before and after implementing the CF program (line 178-182).

(d) It appears that the 2012 data was provided by the FD and the authors collected the 2018 data returning to the same sample plots both in CMBs and SMBs. Is this the case? Then please state clearly in the manuscript.

**Response:** We are grateful to this comment. The current version of the manuscript is revised by indicating the data collection method in both 2012 and 2018 (line 338-340).

(e)How the CMBs and SMBs are spatially related in a CF site. A close-up map of the layout of CMB and SMB blocks or plots in a representative CF site would help clear this up.

**Response:** We are grateful to this comment. The current version of the manuscript is revised by drawing a layout map which shows the CMBs and SMBs spatially related in the CF site (Fig. 3)

(f) The raw data provided seems to only be for CMBs or the treated block there is not a column that indicates control units (SMB) and treated units (CMB), adding to the confusion on this subject. Please make all pertinent data available, in addition to summary statistics, full DID model results as indicated by PLOS Data Policy.

**Response:** The current version of the manuscript is revised by making fully available data indicating control (SMB) and treated unit (CMB).

(g)The fitness and significance of DID linear model needs to be reported (see above on data availability).

**Response:** We are grateful to this comment. The current version of the manuscript is revised by adding related references that show fitness and significance of DID linear model to the current study (line 366-368).

(h) Finally, there a few scattered typos and errors in the text, so a thorough editorial review is also needed for the next revision.

**Response:** We are grateful for the comment. In the current version of the manuscript, the language has been corrected with the help of a professional English editor.

**Reviewer 2**

**Question 1:** Is the manuscript technically sound, and do the data support the conclusions?

**Comment of Reviewer #1:** Partly

**Response:** We are grateful to the second reviewer’s comment. We have modified the original version with technically sound scientific research data that supports the conclusions. Also, our experiment was conducted rigorously, with appropriate controls, replication, and sample sizes.

**Question 2.** Has the statistical analysis been performed appropriately and rigorously?

**Comment of Reviewer #2:** I don’t Know

**Response:** Thank you for comment. The current version of the manuscript is revised by adding related references that shows fitness and significance of DID linear model to the current study (line 372-374).

**Question 3.** Have the authors made all data underlying the findings in their manuscript fully available?

**Comment of Reviewer #2:** Yes

**Response:** Thank you very much for your comment. In the current version of the manuscript, the data underlying the findings described in the manuscript are fully available and indicating control (SMB) and treated unit (CMB).

**Question 4.** Is the manuscript presented in an intelligible fashion and written in Standard English?
**Comment of Reviewer #1:** yes

**Response:** Thank you for the helpful comment.

**Question 5.**

**Comment of Reviewer #2:** The authors present a semi-experimental BACI design study to determine the ecological impacts of community forests (CF) as compared to governmental controlled (FD) forests in Sri Lanka. Their findings indicate that CF showed a significant increase in sapling and seedling density as compared to state-controlled forests, as well as a decrease in certain human disturbance activities. Although the authors present a nice study that I believe would be suitable and of interest to PLOS ONE readers, there are two main issues of concern at present: the lack of detail on the FD sites and greater balance of discussion on the findings in relation to the starting point of the respective forest conditions. Please see my comments and suggestions below.

**Response:** We are grateful to the second reviewer’s beneficial comments. We have modified the original version and have re-submitted a new version by following all of your comments.

INTRODUCTION

**Comment:** P 4 line 71-73, I would suggest that there are stronger citations that might be considered here including Porter-Bolland et al 2012 For Ecol Mang and Bowler et al 2012 Frontiers in Ecol

**Response:** Thank you for your valuable suggestion. The manuscript has been modified by adding suggested references (line 73).

METHODS

**Comment:** Some grammatical errors - e.g. p 8 line 171 use of the word ‘implication’, p 11 line 233 what are agriculture ‘implies’

**Response:** We are grateful for this comment. The manuscript has been modified by correcting the word “implementation” (line173). Agriculture implies are the materials extract from the forest and which use as supportive materials in pandals making in some crops (sticks, ropes).

**Comment:** P 12 line 253, I am a bit confused by your rationale that drought and the loss of crops can be attributed to ‘lead[ing] to rural poverty via deforestation’. By definition, subsistence farmers are poor. Drought and the loss of a large portion of crops could certainly drive a shift in livelihood practices, eg deforestation, driving people further into poverty (or the corollary, be a ‘safety net’ preventing such), but it would not be what made them poor - that is, they already were!

**Response:** Thank you for this comment. The manuscript has been modified by correcting sentence (lines 256-258).

**Comment:** P 12 My first major issue with your paper is the lack of detail on the FD sites. I understand that the CF sites were purposefully selected as I imagine the FD sites were too? There is, however, no indication of such in the methods. Furthermore, I wonder if there were any attempts to ‘match’ or minimally reduce major differences in the CF and FD sites, i.e. proximity to markets, distance to paved road, human population size, or other characteristics. I would also appreciate greater detail on the FD site locations, like that which is provided for the CF sites in Table 1. My ability to evaluate the robustness of your analysis lies in part on my understanding of the validity of the controls, which is a bit elusive at present.

**Response:** Thank you for your valuable comment. In this study we consider the CF site which includes community manage blocks as well as state-managed blocks. The purpose of selecting these 9 CF sites are mentioned in lines 269-280. To make a clear understanding of the distribution of SMB and CMB, a close-up map of the layout of CMB and SMB blocks in a representative CF site (Bambarabedda CF) was included into the new manuscript (Fig. 3).

**Comment:** P 15 There is quite a bit of variation in size of the CFs (50-7,713 ha). How did the FD sites compare? And, I am perplexed why you would sample similarly in areas of such large difference - would this not be particularly important for the large area that may contain rare species or potentially greater variation? I assume there is no issue in identifying the areas where the study occurred? I would suggest modifying your location map to include distinguishing between CF and FD sites.

**Response:** Thank you for your valuable comment. To make a clear understanding of the distribution of SMB and CMB in CF sites a close-up map of the layout of CMB and SMB blocks in a representative CF site (Bambarabedda CF) was included into the new manuscript (Fig. 3).

**Comment:** P 17 I would appreciate a greater understanding of some of your variables, in particular, the number of disease infected trees, pest-attacked trees, land encroachment plots, grazing patches. For example, what constituted ‘encroachment’? Was there a minimum area that had to be disturbed in a defined manner to qualify as such? So data was collected in the same 180 plots first in 2013 and then again in 2018? I think this could be made more explicit earlier on in the methods. With the number of trees lopped or felled, how did you ensure that they were not double counted?

**Response:** We are grateful for this comment. Forest encroachment means to violate the boundary of state land and clearing the land for any other purpose other than forestry (agriculture, construction, mining, digging etc.). As mentioned in lines 267-300, 303-304 in the revised manuscript, the study followed the inventory techniques. According to the literature I followed in the study (especially FD,2014) there are systems of inventorying the trees without double counting. Therefore during the study, no trees were double-counted.

RESULTS

**Comment:** P 21 Paragraph beginning, ‘Indicators of forest. . . ‘ I found your emphasis on tree and seed densities being higher than sapling density confusing with your last sentence that emphasizes increasing sapling density with CF implementation. I would also suggest drawing out the difference between CMB and SMB sites more explicitly.

**Response:** Thank you for your comment. In general, compare to seedling and tree density sapling density is lower in both CMB and SMB. The reason for this is mentioned in line 528-532 in the discussion section of new manuscript. However, the CF program improves the sapling density in CMB but it did not exceed the seedling or tree density.

**Comment:** P 23 lines 465-468 this seems quite anecdotal and perhaps more suited to the discussion?

**Response:** We agree with your comments. The manuscript has been modified by moving the section into discussion part (line 578-582).

**Comment:** P 24, line 473 Delete ‘however’

**Response:** We are grateful for this comment. Current version of manuscript modified by deleting however (line 472).

DISCUSSION

**Comment:** P 25 line 501 It is here that we learn that the CF were established on ‘marginal lands with very few scattered valuable trees’ as well as generally being located closer to villages than SMBs. I would suggest these are rather critical details in the interpretation of your findings as well as things that should likely be stated earlier on in the manuscript. I also wonder how valid it is to compare the impact of CF using SMBs as a comparison if their ecological starting points are so vastly different.

**Response:** Thank you for your comment. As mentioned in line 500-502 in the original manuscript “all forest blocks handed over to the community were marginal lands with very few scattered valuable trees (FD, 2014; Dissanayake, 2013). The layout map (fig 3) shows how CMB and SMB are located in CF sites. In our study, we found that CMBs were closed to the villages and it was the reason for higher human disturbance and less tree density in CMBs. As we found this result in our study we included this section in the discussion part. In addition, we used the DID model to compare the impact of CF. In DID analysis, comparison groups can start at different levels of the outcome (DID focuses on change rather than absolute levels) (Angrist and Pischke, 2008). This is the main strength of the DID which makes it more suitable for the current study.

Angrist J, Pischke JS. Mostly Harmless Econometrics. Princeton University Press, NJ 2008.

**Comment:** Nonetheless, I find aspects of your results quite compelling and insightful. In particular, the reduction in invasives and fire occurrence are no doubt due in large part to community effort. åSimilarly, that seedling and sapling densities have increased and certain human activities, decreased. I would suggest greater attention to your selection of the FDs and being more upfront about the differences in CMBs versus SMBs will strengthen the paper and facilitate its publication.

**Response:** We modified the manuscript by drawing the layout of CMB and SMB and adding the main differences of the state forest which are subjected to implement CF program and which are not (Fig. 3, line 178-182).
