# Supplementary material for: Impacts of community forestry on forest condition: Evidence from Sri Lanka’s intermediate zone
Source: PLoS One. 2020 Sep 30;15(9):e0239405. doi: 10.1371/journal.pone.0239405 (PMC7526917; doi:10.1371/journal.pone.0239405)
Supplement: Attachment — Submitted filename: Response to Reviewers.docx [file pone.0239405.s006.docx]

**Rebuttal letter**

**Academic Editor Comment**

**Comment 1**

In lines 593-595 you state that forest boundaries were clearly marked and a fire-belt constructed in the CMBs as a result of the CF program. However, in newly added text (lines 170-174) you indicate that the FD did those things, presumably once the blocks were selected for the CF program. It’s important to be clear about that. In lines 601-605, you assert that the CF program reduced the occurrence of forest fires in both CMBs and SMBs. This conflicts somewhat with Table 5 – yes, the DID coefficient for fire is negative and significant, but the values of the fire presence variable for the SMBs are the same before and after (0.23). So, did the CF program really make a difference in terms of fire in the SMBs? (On the other hand, it seems pretty conclusive for the CMBs.)

**Response 1**

We are grateful for this comment. As mentioned in the manuscript FD established a fire belt in selected forest areas based on the previous recodes of fire occurrence however, due to high labor cost and limited resources FD unable to maintain the fire belt throughout the year. Once the fire belt opens by FD it will reopen in next year. This is not effective in controlling fire, because most of the grasses (cause for fire spreading) are growing fast and cover the fire belt. Due to this reason, FD encourages CF members to established and maintain a fire belt around CF sites. The current version of the manuscript modified by clarifying these issues. (Lines 202-205)

Furthermore, as indicated by our results, the occurrence of fire in both CMBs and SMBs reduced due to the implementation of the CF program. However, if the fire origin from inside the state forest or another adjacent settlement other than the CF area, those fire cannot be controlled by the fire belt established by the FD. We modified the current version of the manuscript by indicating these reasons (lines 583-584).

**Additional Editor Comments**

**Comment 1**

Line 78 – delete comma after “program”

**Response**

We are grateful to the Additional Editors’ beneficial comments. The current version of the manuscript has been modified by deleting the comma after “program” (line 78).

**Comment 2**

Line 79 – change “subsistent” to “subsistence”

**Response2**

We are grateful to this comment. The current version of the manuscript has been modified by changing “subsistent” to “subsistence” (line 79).

**Comment 3**

Line 85 – insert comma after “sacred”

**Response3**

We are grateful to this comment. The current version of the manuscript has been modified by inserting a comma after “sacred” (line 85).

**Comment 4**

Line 102 – delete “very”

**Response 4**

We are grateful to this comment. The current version of the manuscript has been modified by deleting “very” (line 102).

**Comment 5**

Line 150 – spell out non-timber forest products at this first use

**Response5**

Thank you for the helpful comment. The current version of the manuscript has been modified by spelling out non-timber forest products (line 150).

**Comment 6**

Line 163 – “transfers” instead of “transfer”

**Response 6**

Thank you for the helpful comment. The current version of the manuscript has been modified by using “transfers” instead of “transfer” (line 163).

**Comment 7**

Line 166 – “state-managed” (insert dash)

**Response 7**

Thank you for the helpful comment. The current version of the manuscript has been modified by inserting dash in “state-managed” (line 166).

**Comment 8**

Lines 170-174 – The new text needs a slight rewrite, something like: “In Sri Lanka, forests entered into the CF program were previously state-managed forests. Under state management, the majority of natural forests were protected, and forestry operations such as extraction (e.g. timber) were restricted. However, boundary demarcation and fire-belt establishment were conducted by FD using forest laborers. Furthermore, when the CF program was implemented in forest blocks, the adjacent state-managed forest blocks remained subject to the same FD management activities as before the CF program.” In addition, I think this new text might fit better around line 202.

**Response 8**

Thank you for the helpful comment. The current version of the manuscript has been modified by rewriting the line and we transferred those lines into 202 in the old manuscript (lines 197-202)

**Comment 9**

Line 185 – “impacts” instead of “impact”

**Response 9**

We are grateful to this comment. The current version of the manuscript has been modified by inserting “impacts” instead of “impact” (line 180).

**Comment 10**

Line 204 – “lands” instead of “land”

**Response 10**

We are grateful to this comment. The current version of the manuscript has been modified by inserting “lands” instead of “land” (line 207).

**Comment 11**

Line 214 – comma after “Climate”

**Response 11**

We are grateful to this comment. The current version of the manuscript has been modified by inserting a comma after “Climate” (line 218).

**Comment 12**

Line 219 – insert “a” before “low”

**Response 12**

We are grateful to this comment. The current version of the manuscript has been modified by inserting “a” before “low” (line 223)

**Comment 13**

Line 252 – rewrite: “…the highest numbers of people who were below the poverty line were…”

**Response 13**

Thank you for the helpful comment. The current version of the manuscript has been modified by rewriting the line (line 240).

**Comment 14**

Line 286 – delete comma after “sites”

**Response 14**

We are grateful to this comment. The current version of the manuscript has been modified by deleting comma after “sites” (line 270).

**Comment 15**

Line 352 – rewrite: “…encroachment plots (Table 2) is a count of the number of locations that violate…”

**Response 15**

Thank you for the helpful comment. The current version of the manuscript has been modified by rewriting the line (line 335).

**Comment 16**

Table 5 – “Lopped” instead of “Loped”; capitalize the “Before” and “After” columns for the CMBs

**Response 16**

Thank you for the helpful comment. The current version of the manuscript has been modified by correcting Lopped” instead of “Loped”; capitalizing the “Before” and “After” columns for the CMBs (Table 5).

**Comment 17**

Line 497 – “CMBs” instead of “CMB”

**Response 17**

We are grateful to this comment. The current version of the manuscript has been modified by inserting “CMBs” instead of “CMB” (line 475).

**Comment 18**

Line 534 – delete comma after “income”

**Response 18**

We are grateful to this comment. The current version of the manuscript has been modified by deleting comma after “income” (line 512).

**Comment 19**

Line 559 – “state-managed” (insert dash)

**Response 19**

Thank you for the helpful comment. The current version of the manuscript has been modified by inserting dash in “state-managed” (line 537).

**Comment 20**

Line 560 – rewrite: “…in seedling and sapling density…”

**Response 20**

Thank you for the helpful comment. The current version of the manuscript has been modified by rewriting the line (line 538).

**Comment 21**

Line 568 – “state-managed” (insert dash)

**Response 21**

Thank you for the helpful comment. The current version of the manuscript has been modified by inserting dash in “state-managed” (line 546).

**Comment 22**

Line 584 – “results” instead of “result”

**Response 22**

Thank you for the helpful comment. The current version of the manuscript has been modified by inserting “results” instead of “result” (line 562).

**Comment 23**

Line 638 – “community-based” (insert dash)

**Response 23**

Thank you for the helpful comment. The current version of the manuscript has been modified by inserting dash in “community-based” (line 617).

**Comment 24**

Line 651 – delete comma after “species”

**Response 24**

Thank you for the helpful comment. The current version of the manuscript has been modified by deleting comma after “species” (line 630).
